# Detection of human and rodent 5-HT_3B _receptor subunits by anti-peptide polyclonal antibodies

**DOI:** 10.1186/1471-2202-7-27

**Published:** 2006-03-29

**Authors:** David C Reeves, Sarah CR Lummis

**Affiliations:** 1 Department of Biochemistry, University of Cambridge, Cambridge CB2 1QW, UK; 2 Department of Physiology and Biophysics, Albert Einstein College of Medicine, Bronx, NY, 10461, USA

## Abstract

**Background:**

The 5-HT_3 _receptor is a member of a neurotransmitter-gated ion channel family which includes nicotinic acetylcholine, GABA_A_, and glycine receptors. While antibodies specific for the 5-HT_3A _receptor subunit are plentiful, and have revealed a wealth of structural and functional information, few antisera exist for the detection of 5-HT_3B _receptor subunits. Here we describe the generation and characterisation of a rabbit polyclonal antiserum that specifically recognises 5-HT_3B _receptor subunits

**Results:**

Immunization of a rabbit with a 20-mer peptide, corresponding to the N-terminus of the human 5-HT_3B _receptor subunit, generated serum with polyclonal antibodies from which an IgG fraction was purified, yielding pAb77. The antibodies were shown to label 5-HT_3B _receptor subunits in transfected human embryonic kidney cells and rodent tissues using Western blots. Immunocytochemistry using pAb77 on these cells showed that 5-HT_3B _receptor subunits do not reach the plasma membrane in the absence of 5-HT_3A _receptor subunits. Immunohistochemical analysis of rat brain sections showed pAb77 immunoreactivity in distinct populations of cells in the hippocampus.

**Conclusion:**

We have demonstrated that pAb77 antibodies specifically label native and recombinant 5-HT_3B _receptor subunits with high affinity and specificity. The antibody was shown to be useful for the determination of both receptor trafficking and also mapping 5-HT_3B _receptor subunit expression in the CNS.

## Background

The 5-HT_3 _receptor is a pentameric neurotransmitter gated ion channel that acts as a molecular switch at synapses [1]. 5-HT_3 _receptor antagonists are used clinically for the prevention of emesis, and may be useful in the treatment of irritable bowel syndrome and alcoholism [2]. Interestingly, mice lacking the 5-HT_3A _receptor subunit were recently shown to possess an anxiolytic phenotype [3], although therapeutic potential in humans appears limited [4].

Five 5-HT_3 _receptor subunits have been cloned to date [5-7], although only two (5-HT_3A _and 5-HT_3B_) have been shown to exist in functional receptors. Homomeric 5-HT_3A _receptors are notable for their sub-pS single channel conductance [8], but co-expression with 5-HT_3B _receptor subunits increases the conductance to more than 20 pS [9]. The pharmacological properties of the heteropentamer are largely unchanged compared with the homopentamer [10,11], although the Ca^2+ ^permeability of the heteropentamer may be ablated [12] and recovery from desensitization quickened [13]. Additionally, rodent 5-HT_3A/B _heteromers have reduced sensitivity to picrotoxin [14], and channel gating enhancement by some anaesthetics (especially halothane and chloroform) is markedly attenuated [15]. The stoichiometry of recombinant 5-HT_3A/B _heteropentamers has recently been determined as BBABA [16] but the stoichiometry of native 5-HT_3 _receptors remains to be confirmed.

While expression of 5-HT_3B _receptor subunits has been demonstrated in the peripheral nervous system [17], controversy remains over their presence in the central nervous system (CNS) [18]. In particular, an antiserum previously generated which recognizes 5-HT_3B _receptor subunits (AP86/3, [19]) labelled cells in the rat hippocampus, despite in situ hybridisation and RT-PCR data suggesting absence of the subunit [20,21]). On the other hand, 5-HT_3 _receptor expression in the brain is widespread [22] and 5-HT_3B _receptor subunit mRNA has been detected in whole brain lysates [6]. It is possible that the 5-HT_3B _receptor subunit, if expressed in the brain, could be targeted to small, discrete populations of cells. The availability of sensitive and selective antisera could help to clarify these points.

Here we describe the generation, purification and characterization of polyclonal antibodies (pAb77) to a peptide corresponding to a region of the extracellular domain of the 5-HT_3B _receptor subunit.

## Results and discussion

### Immunoreactivity of antisera for 5-HT_3B _receptor subunits

Two rabbits (numbered 6376 and 6377) were immunised with coupled peptide every two weeks for a period of approximately two months. Test-bleeds were taken 7–10 days after immunisations. Western blots were used to test each antiserum by probing membranes electroblotted with separated crude lysates of human embryonic kidney (HEK)293 cells stably expressing the human 5-HT_3B _receptor subunit (not shown). No consistent immunoreactivity was observed for sera from rabbit 6376 or pre-immune serum from either animal. At week 7 and subsequent bleeds, serum from Rabbit 6377 showed a consistent strong immunoreactivity for a 55–60 kDa protein. The predicted molecular weight of human 5-HT_3B _receptor subunits without post-translational modifications is 50.3 kDa. We inferred that this immunoreactivity represented labelling of the 5-HT_3B _receptor subunit.

**Figure 1:**
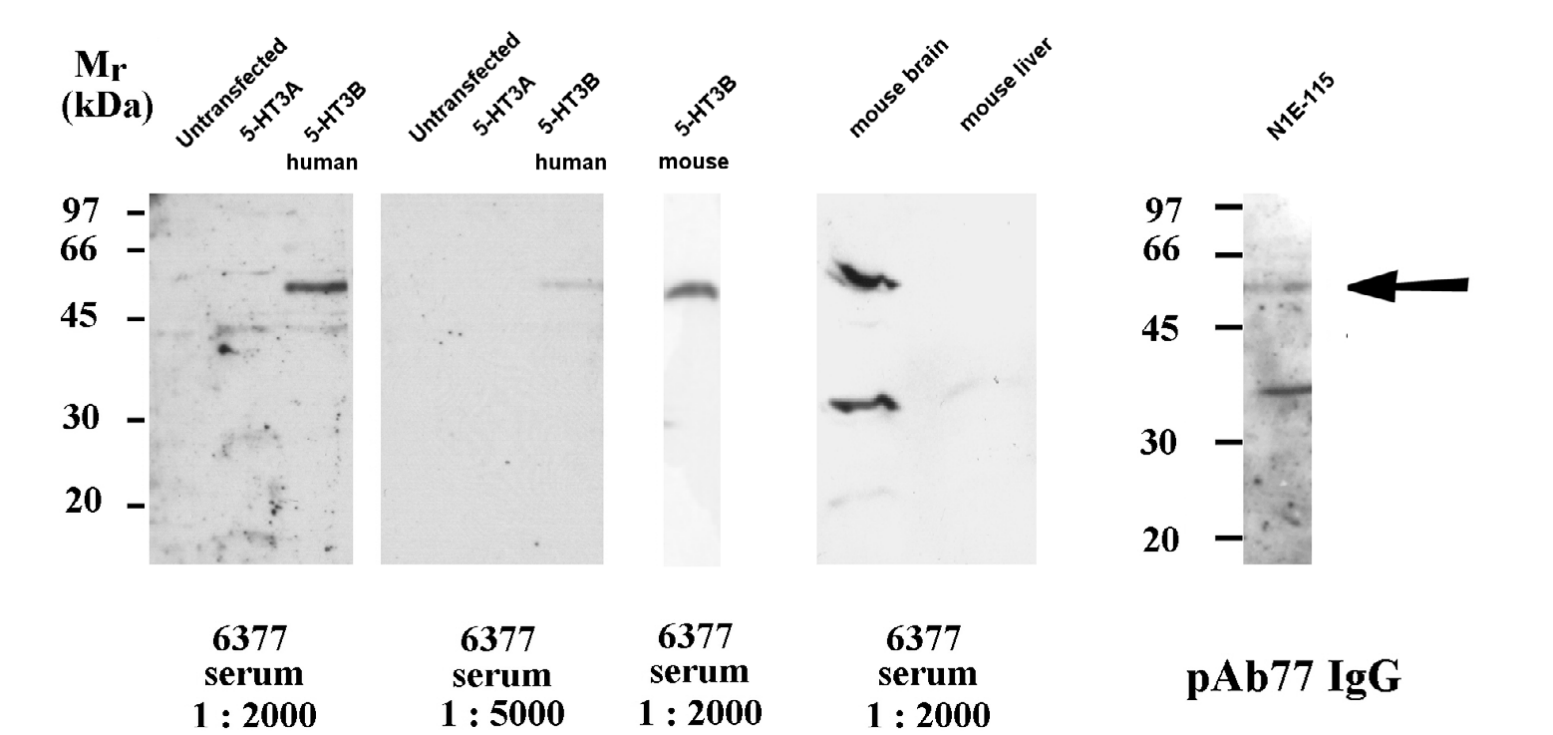
**Western blots of lysates from untransfected HEK293 cells and HEK293 cells expressing mouse 5-HT_3A _or 5-HT_3B _receptor subunits, or human 5-HT_3B _receptor subunits**. Blotted membranes were hybridised with dilutions of 6377 serum or purified pAb77 IgG fraction as the primary antibody. Data are typical of three or more independent experiments. The arrow indicates the position of 5-HT_3B _receptor subunits. Western blots using lysates from mouse tissues and N1E-115 cells are shown on the right hand side.

Serum 6377 was tested at several different dilutions against homogenates of HEK293 cells stably expressing 5-HT_3 _receptor subunits. Western blots revealed non-specific labelling of several bands at a dilution of 1:100 of 6377 serum (not shown), but dilutions of 1:2,000 to 1:5,000 labelled a single protein band at 55–60 kDa from lysates of cells expressing either human or mouse 5-HT_3B _receptorsubunits (Figure 1). No labelling was seen with lysates of untransfected cells, with cells expressing 5-HT_3A _receptor subunits, or following pre-incubation of 6377 serum for 15 min with 5 mg/ml of the synthetic peptide (data not shown).

Purified IgG fraction (pAb77) was tested by Western blot for its ability to recognise 5-HT_3B _receptor subunit protein as above. Purified IgG was compared with crude 6377 serum by applying each solution separately as the primary antibody (dilution 1:1000) to nitrocellulose membranes blotted with lysates of HEK293 cells expressing 5-HT_3A _or 5-HT_3B _receptor subunits, untransfected cells, and lysates of N1E-115 cells (Figure 1). In common with the crude serum, pAb77 did not label proteins from untransfected cells or those expressing 5-HT_3A _receptor subunits. pAb77 IgG was shown to retain its capacity to specifically label a 55–60 kDa protein in cells expressing human 5-HT_3B _receptor subunits, with a substantial decrease in non-specific background labelling.

We infer that the pAb77 antibodies are both specific and sensitive for 5-HT_3B _receptor subunits. The previously reported 5-HT_3B _specific antiserum, AP86/3 [19], detected a band of 50–60 KDa at a concentration of 0.14 μg/ml. Our new antiserum appears equally or perhaps more sensitive: We observed labelling at dilutions of 1: 10,000 crude sera (~0.1 μg/ml), with strong bands using 1:5000 pAb77 (~0.2 μg/ml). This latter allowed detection of 5-HT_3B _receptor subunits in homogenates of N1E-115 cells, which express heteromeric rodent 5-HT_3 _receptors [23]. Here pAb77 recognised a 55–60 kDa protein, corresponding to the 5-HT_3B _receptor subunit.

### Visualisation of 5-HT_3B _receptor subunits by indirect immunofluorescence

**Figure 2:**
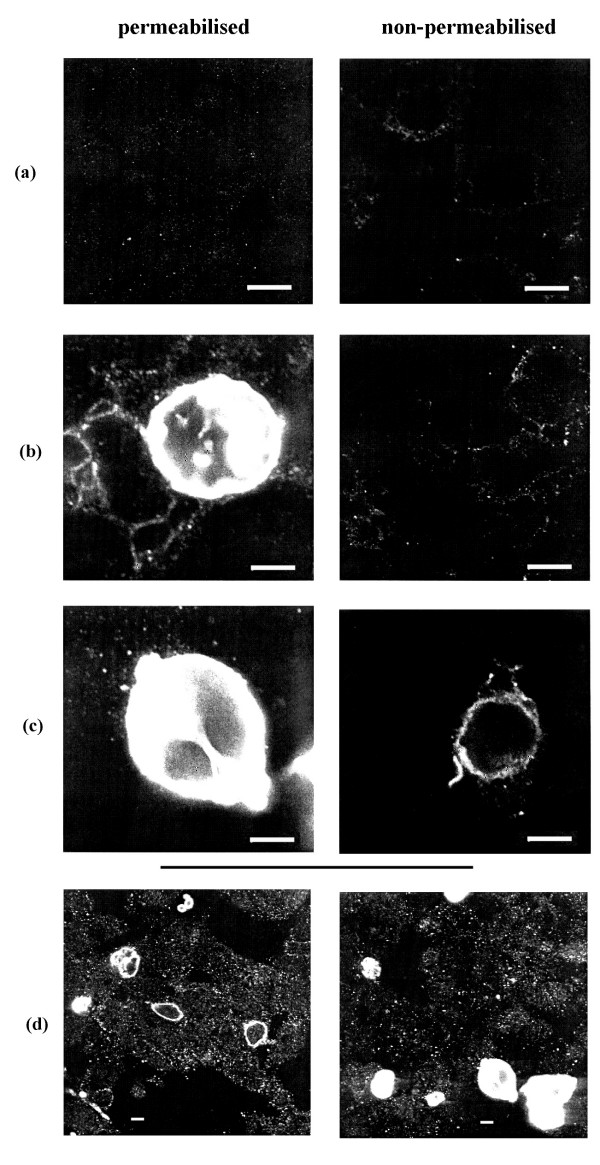
**Immunolabelling by pAb77 of 5-HT_3A _and 5-HT_3B _receptor subunits expressed in HEK293 cells**. Confocal images of HEK293 cells fixed with 4% paraformaldehyde probed with immune sera and visualised by indirect immunofluorescence (FITC). (a) transfection of h5-HT_3B _probed with pre-immune sera (1:1000); (b) transient transfection of h5-HT_3B _probed with pAb77 (1:1000); (c) transient transfection of h5-HT_3B _and m5-HT_3A _probed with pAb77 (1:1000); (d) permeabilised cells (both panels) transiently transfected with either (left panel) m5-HT_3A _subunit cDNA, and probed with pAb120 antibody (1:300) or (right panel) m5-HT_3A _and h5-HT_3B _cDNA and probed with pAb77. Scale bars represent 10 μM.

HEK293 cells were transiently transfected with 5-HT_3A _or 5-HT_3B _receptor subunits alone or in combination, and labelled with either pre-immune serum or final bleed polyclonal serum from rabbit 6377. Typical data is shown in Figure 2. No labelling was observed with pre-immune serum from rabbit 6377 (Fig. 2a). Strong labelling was obtained from permeabilized cells expressing either 5-HT_3B _receptor subunits alone (Fig. 2b) or in combination with 5-HT_3A _receptor subunits (Fig. 2c). Labelling was also seen around the cell membrane of some non-permeabilised cells transfected with both 5-HT_3A _and 5-HT_3B _receptor subunits (Fig. 2c, right panel). This suggests that at least some 5-HT_3B _receptor subunits are expressed on the surface in the presence of 5-HT_3A _receptor subunits, although the majority of the 5-HT_3B _receptor subunits remain intracellular. The presence of 5-HT_3A _receptor subunits on the surface of cells transfected with both 5-HT_3A _and 5-HT_3B _receptor subunits was verified by labelling with pAb120, an antisera selective for 5-HT_3A _receptor subunits [24] (Figure 2d).

The extracellular location of the antisera binding site means that it can be used to probe the location of 5-HT_3B _receptor subunits on the cell membrane. Both 6377 serum and pAb77 IgG were found to produce specific labelling of human 5-HT_3B _receptor subunits expressed in permeabilised HEK293 cells, with low background labelling. Specific labelling of non-permeabilised cells, however, was only seen in the presence of 5-HT_3A _receptor subunits (Fig. 2c), suggesting that these are required for efficient expression of 5-HT_3B _receptor subunits on the surface. Thus our immunofluorescence data concur with previous reports that 5-HT_3B _receptor subunits remain intracellular unless co-expressed with 5-HT_3A _[29,26].

**Figure 3:**
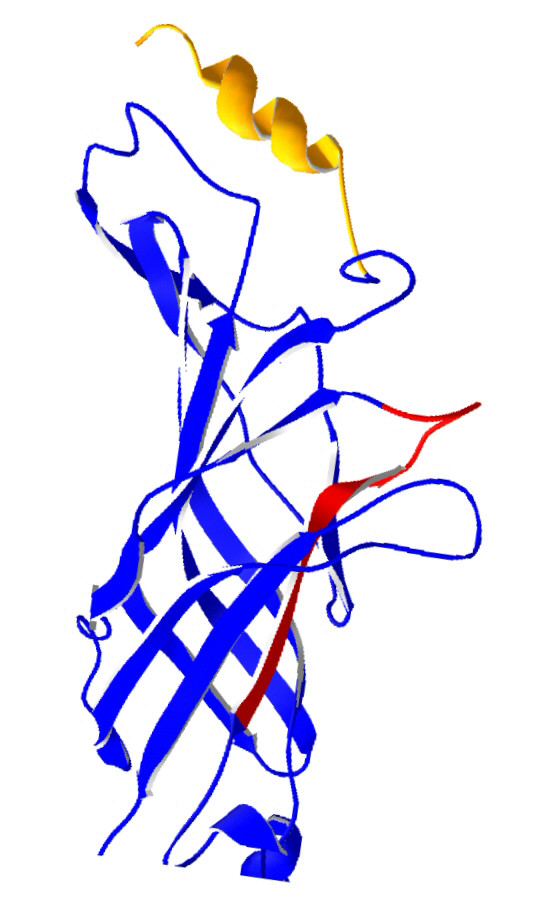
**Model of the 5-HT_3B _receptor subunit extracellular domain**. based on the structure of the nicotinic acetylcholine receptor, showing the location of peptides corresponding to those used as immunogens for pAb77 (yellow) and AP86/3 (red).

As the peptide used to generate the polyclonal antibodies corresponds to part of the N-terminal region of the 5-HT_3B _receptor subunit, it corresponds to part of the extracellular domain. Using the recently published structure of the homologous nicotinic acetylcholine receptor [25], we can identify the likely region to which it binds (Fig 3). The peptide used as an immunogen in this study corresponds to the α-helix at the tip of the extracellular domain. This model also reveals why pAb77 may be more sensitive than AP86/3 in non-denaturing immunostaining experiments, as the region against which the latter was raised appears to be partly buried in the folded subunit.

### Immunohistochemical localisation of 5-HT_3B _subunits in rat brain

The immunogenic peptide from human 5-HT_3B _receptor subunits used in this study is 58% identical to the mouse 5-HT_3B _sequence in that region, and for 89% of the residues the charge and polarity are conserved. The sequences of the cloned 5-HT_3B _receptor subunits from mouse and rat in the region of the peptide used in this study are identical [23]. Western blots of HEK293 cells transfected with mouse 5-HT_3B _receptor subunits or mouse brain (but not liver) homogenates (Figure 1) showed that the polyclonal antibodies specifically labelled a band of approximately 55–60 kDa corresponding to 5-HT_3B _receptor subunits. A further band, of M_r _approximately 35 kDa was detected by pAb77 in mouse brain or N1E-115 cell homogenates. An extra 35 kDa band has previously been observed with polyclonal antibodies raised against the 5-HT_3A _receptor subunit, and considered to represent a degradation product [24], although differing glycosylation state or a splice variant are also possibilities.

**Figure 4:**
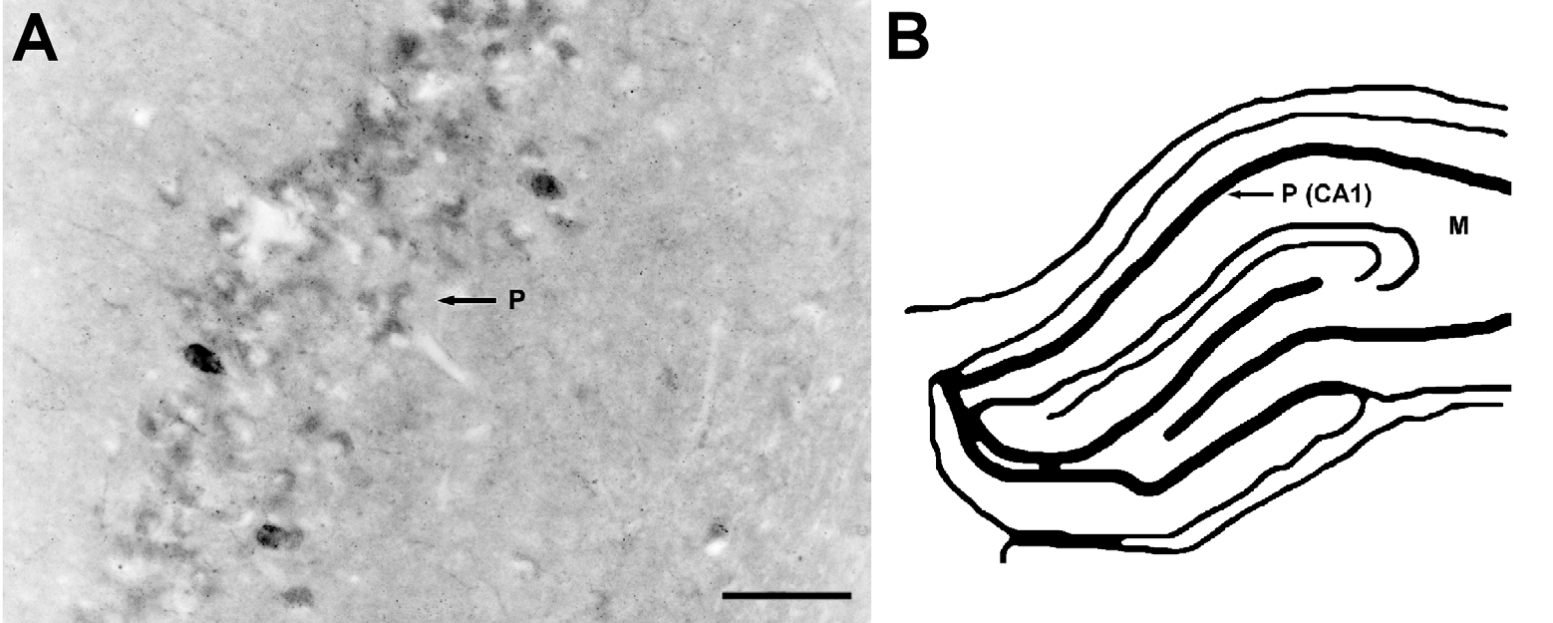
**Immunohistochemical labelling of rat hippocampus with pAb77**. **(A)**. Typical results from immunoperoxidase-linked visualisation of pAb77 (1:5000) binding to rat hippocampal cryostat sections. Note the strong labelling of certain cells in the pyramidal cell layer (P). Scale bar represents 50 μM. **(B) **Schematic drawing of a coronal section of rat hippocampus. P = pyramidal layer, M = molecular layer.

The antibodies were used in further immunohistochemical experiments to determine the distribution of 5-HT_3B _receptor subunits in rat brain and thereby expand the mapping of 5-HT_3A _receptors previously described [27]. Sections of rat hippocampus were probed with either final bleed serum from rabbit 6377 or pAb77, and the optimal dilution was determined to be 1:1,000–1:5,000. The pattern of labelling was not significantly different between crude 6377 serum and purified pAb77. The data show a general light labelling of neurons plus a stronger staining of certain cells in the pyramidal and molecular layers of the hippocampus (Figure 4).

Similar results were obtained with pAb77 in rat hippocampus as were previously demonstrated for the 5-HT_3A _specific antibody, pAb120, generated in a previous study [24], except that no axonal labelling was seen with pAb77. The lack of axonal labelling by pAb77 could reflect a difference in the sub-cellular distribution of the subunits, although further experiments are required to confirm this. In situ hybridisation using riboprobes has revealed that different cell types in the rat spinal cord can express different combinations of 5-HT_3 _receptor subunits. 70% of cells in the rat spinal cord express 5-HT_3A _receptor subunits, but only 50% of those express 5-HT_3B _receptor subunits, while 91% of neurons that express 5-HT_3B _receptor subunits also express 5-HT_3A _[28]. Additionally, it has been shown that differentiation of rat PC-12 cells induced by NGF increases 5-HT_3B _receptor subunit expression [23]. Our data, and that utilizing AP86/3 serum [19], which also produced labelling of rat hippocampal sections, support the presence of 5-HT_3B _receptor subunits on certain CNS neurons, and suggests that 5-HT_3B _receptor subunits are present at low concentrations and are highly localised in discrete populations. The presence of the 5-HT_3B _receptor subunit in the CNS may in fact be neuroprotective, as heteropentameric 5-HT_3 _receptors have a very low Ca^2+ ^permeability relative to homopentameric 5-HT_3A _receptors, which precludes the possibility of Ca^2+^-induced neurotoxicity in the CNS being due to 5-HT_3A/B _heteropentameric receptors [12].

## Conclusion

Polyclonal antiserum raised against the peptide, ^27^PQDSALYHLSKQLLQKYHK^46^C, was shown to specifically bind 5-HT_3B _receptor subunits with high affinity, allowing the labelling of human and rodent 5-HT_3B _receptor subunit protein in Western blot, immunocytochemical and immunohistochemical procedures. It was found that the majority of 5-HT_3B _receptor subunits, when expressed alone, do not reach the cell surface, but that co-expression of 5-HT_3A _with 5-HT_3B _receptor subunits permits expression of 5-HT_3B _receptor subunits in the plasma membrane, as reported previously [29]. Receptor subunits were observed in rat hippocampal slices, but only in a small percentage of cells, suggesting they exist in the CNS in low abundance and may be highly localised. This new pAb77 antiserum should help to clarify the location and function of both native and recombinant 5-HT_3B _receptor subunits.

## Methods

### Design and synthesis of antigenic peptide

The peptide sequence of the human 5-HT_3B _receptor subunit was analysed with ANTHEPROT v5.2, [30] and regions of the protein predicted to have a high probability of antigenicity were identified. A second program, ANTIGENIC, from the EMBOSS molecular biology software suite was used to determine candidate peptides [31]. As very short peptides may decrease antigenicity, candidates of less than thirteen residues were discarded. Peptides with more than fifteen amino acids could increase the chance of antibodies being generated against more than one area of charge density [32] therefore these were also discarded. Candidates not in the predicted N-terminal domain were eliminated to allow extracellular labelling by the antibody. The final choice of the peptide ^27^PQDSALYHLSKQLLQKYHK^46^C, was made because it contains a high proportion of charged residues and is in a similar region just C-terminal to the signal peptide to the peptide that was previously used successfully to generate antibodies selective for the 5-HT_3A _receptor subunit [24]. The final cysteine residue was added to the sequence to allow coupling to the carrier. The peptide sequence was checked by BLAST alignment against the non-redundant sequence database and at lower stringency (E = 100) against all available LGIC subunit sequences [33]. The peptide was found to be unique to 5-HT_3B _receptor subunit protein. The peptide was synthesised by Research Genetics (Huntsville, AL).

### Preparation of antigenic peptide for injection

The immunogenic peptide was coupled to keyhole limpet haemocyanin (KLH) by standard methods [34]. Briefly, 1 ml of a 10 mg/ml solution of KLH in PBS (pH 6.0) was mixed with 1 mg *m*-maleimidobenzoic acid (MBS) for 30 min. Coupled KLH-MBS was purified using a Sephadex G-25 column equilibrated to pH 7.4. The peptide was reduced with 0.2 M sodium borate for 10 min followed by neutralisation with 1 M HCl. 5 mg of reduced peptide was dissolved in the KLH-MBS solution and stirred for 1 h at room temperature. The coupled peptide was stored at -20°C until required. Two male, New Zealand White, rabbits were injected subcutaneously with 1 mg conjugated peptide, mixed with Freund's complete adjuvant. At monthly intervals, for three months, rabbits were given booster injections with 1 mg of conjugated peptide mixed with Freund's incomplete adjuvant. A harvest bleed was taken at week 9.

### SDS PAGE and Western blotting

Lysates of HEK293 cells transiently or stably expressing 5-HT_3 _receptor subunits, or N1E-115 cells, were prepared by freezing as previously described [24,35]. For mouse tissues, 200 mg tissue was macerated with a needle in 0.5 ml of extraction buffer containing 200 mM Tris-HCl pH 8.0, 250 mM NaCl, 25 mM EDTA and 0.5% SDS. The solution was incubated at 50°C for 30 min and centrifuged at 4°C for 5 min at 14 000 g. Samples of the supernatant were mixed with an equal volume of electrophoresis sample buffer (2% SDS, 100 mM dithiothreitol, 60 mM Tris-HCl pH 6.8, 0.05% bromophenol blue) and heated at 95°C for 5 min. They were then centrifuged at 21 000 g for 5 min and 15 μl loaded per lane on SDS-polyacrylamide gels (10% – 12%) using the Mini-Protean 3 system (BioRad). Semi-dry electrophoretic transfer was used to electroblot the separated proteins onto Hybond-ECL nitrocellulose membranes (Amersham-Pharmacia) at 200 mA for 60–90 min (Biometra, Germany). Membranes were stained using Ponceau S for 5 min to verify complete transfer of proteins from the gel. The membranes were incubated in PBS with 5% dried milk powder (Marvel) for 1 h to block non-specific binding sites and then incubated in primary antibody solution in PBS/milk for 1 h with continuous agitation at room temperature. Membranes were washed four times with PBS and then incubated with secondary antibody (a 1:2000 dilution in PBS/milk of goat anti-rabbit antibody linked with horseradish peroxidase, Sigma, MO). Labelling was visualised using the ECL+ chemiluminescence system (Amersham-Pharmacia). For some blots, Tween 20 was added to the PBS solutions at 0.05% to reduce background labelling. Chemiluminescence was recorded by 10–360 s exposures of Hyperfilm-ECL developed using an automatic film developer (X-Omat; Kodak, US).

### Purification of IgG fraction from immunoreactive serum

In order to decrease non-specific staining seen with the crude serum from rabbit 6377, an immunoglobulin fraction was affinity purified using a Protein A column. An ImmunoPure Immobilised Protein A column (Pierce, Rockford, IL) was equilibrated with 5 ml Binding Buffer (Pierce). 1 ml of the 6377 final bleed serum was diluted with 1 ml Binding Buffer and added to the column. 10 volumes of Binding Buffer were added to the column and binding was monitored by measuring the absorbance at 280 nm of 1 ml fractions of the flow-through. 5 ml Elution Buffer was added to the column and the eluate containing the IgG was collected. The eluate was concentrated using a Centriprep-10 column (Millipore) following the manufacturer's instructions to give a final volume of 700 μl (in 1 M Tris-HCl pH 8.0). The purified polyclonal antibody is referred to here as 'pAb77'.

### Immunocytochemical visualisation of 5-HT_3 _receptors

HEK293 cells were grown on 22 mm glass cover slips and washed three times with Tris-buffered saline (TBS: 0.1 M Tris-HCl pH 7.4, 0.9% NaCl) fixed using 4% paraformaldehyde and then incubated in the appropriate dilution of pAb77 antibody (1:100–1:2000) for 1 h at room temperature. After three further washes with TBS, biotinylated anti-rabbit IgG (Vector, CA) was added at 1:200 in TBS and the cover slips incubated for 1 h at room temperature and washed as before. Fluorescein isothiocyanate avidin D (Vector) was added at a dilution of 1:200 in TBS with incubation for 1 h at room temperature and washed as above. The cover slips were mounted in Vectashield mounting medium (Vector) and immunofluorescence observed using a Nikon epifluorescence microscope. Photomicrographs were taken using constant exposure times of 50 s on Fuji RealA colour film (ASA 400) using a Nikon camera attached to the microscope. Negatives were printed by AM Photographic (Cambridge, UK) with image brightness equalised to that of the negatives. Confocal images were obtained for some preparations (BioRad-MRC600).

### Immunohistochemical visualisation of 5-HT_3 _receptors

Immunohistochemistry on tissue sections from rat brain was performed using standard immunofluorescence and immunoperoxidase procedures [27]. All procedures were performed at room temperature unless otherwise stated. Male Wistar rats (200–250 g) were deeply anaesthetized and transcardially perfused with 100 ml 0.9% saline followed by 400 ml 4% paraformaldehyde in 0.1 M phosphate buffer. Brains were removed, postfixed for 2 h and cryoprotected overnight in 15% sucrose in 0.1 M phosphate buffer. Cryostat slide-mounted sections (15 μm), or free floating sections cut on a Vibratome (40 μm), were incubated for 1 h in 10% normal goat serum followed by incubation with either crude 6377 serum (1:500–1:10,000) or purified pAb77 IgG (1:500-1:500) in phosphate buffered saline (PBS) containing 0.2% Triton X-100.

For immunofluorescence, sections were incubated for 48 h in primary antibody then washed in PBS and incubated with tetramethylrhodamine-labelled donkey anti-rabbit IgG (1:200) for 3 h. Sections were washed and mounted in PBS:glycerol (1:3) containing 2.5% 1,4-diazobicyclo (2,2,2)- octane to reduce fading. Sections for the immunoperoxidase procedure were incubated for 48 h in primary antibody and 2 h in biotinylated goat anti-rabbit antiserum (1:400) followed by 2 h in avidin-biotin complex (ABC, Vectastain Kit, Vector Laboratories). Immunoreactivity was detected using the nickel-intensified diaminobenzadine method.

## Abbreviations

5-HT: 5-hydroxytryptamine; CNS: central nervous system; HEK: human embryonic kidney; KLH: keyhole limpet haemocyanin; MBS: *m*-maleimidobenzoic acid; TBS: Tris-buffered saline

## Authors' contributions

DCR carried out all experimental procedures except for certain Western blots and the immunofluorescence experiments, which were performed by SCRL.
